# Microsatellite Stable Colorectal Tumours in Patients with Lynch Syndrome: A Case Report and Systematic Review Analysing Clinical Features and Implications for Immunotherapy

**DOI:** 10.1007/s12029-025-01203-1

**Published:** 2025-03-25

**Authors:** Fani Kapoulitsa, Davide Mauri, Konstantinos K. Tsilidis, Konstantinos Katsanos, Eleni Timotheadou, Maria Smaragdi Vlachou, Konstantinos Kamposioras

**Affiliations:** 1 Department of Medical Oncology, University of Ioannina, Ioannina, Greece; 2 Department of Hygiene and Epidemiology, University of Ioannina, Ioannina, Greece; 3 Department of Epidemiology and Biostatistics, School of Public Health, Imperial College London, London, UK; 4 Department of Gastroenterology, University of Ioannina, Ioannina, Greece; 5 Department of Medical Oncology, Aristotle University of Thessaloniki, Papageorgiou Hospital, Thessaloniki, Greece; 6 Department of Medical Oncology, The Christie NHS Foundation Trust, Manchester, UK; 7 Division of Cancer Sciences, Faculty of Biology, Medicine and Health, The University of Manchester, Manchester, UK

**Keywords:** Lynch Syndrome, Immune checkpoint inhibitors (ICI), DNA mismatch repair (MMR), Microsatellite stability (MSS), DMMR/MSS

## Abstract

**Purpose:**

Lynch syndrome is an autosomal dominant genetic disorder associated with early-onset colorectal cancer (CRC), endometrial cancer and other malignancies. This condition is defined by deficient DNA mismatch repair and high microsatellite instability (dMMR/MSI-high), exhibiting a substantial response to immunotherapy. However, microsatellite-stable (MSS) tumours may infrequently occur in individuals with Lynch syndrome. Our aim was to evaluate the efficacy of immunotherapy in patients with Lynch Syndrome and dMMR/MSS colorectal cancer.

**Methods:**

A systematic review of the literature in medical databases, major related conferences and relevant oncology journals was conducted to identify the available evidence. Medical records from the Medical and Clinical Oncology Department of the University Hospital of Ioannina were also reviewed.

**Results:**

Four cases of MSS colorectal cancer associated with Lynch syndrome and MSH6 germline mutation were identified. Three of these four patients were treated with immune checkpoint inhibitors. Two patients with metastatic disease experienced disease progression, but one patient who received neoadjuvant immunotherapy achieved a partial response. All four patients were diagnosed with colorectal cancer in ages younger than 52 (16–51 years old).

**Conclusion:**

MSS CRC tumours in patients with Lynch syndrome is an infrequent phenomenon and under-represented in the literature. The limited efficacy of immune checkpoint inhibitors is highlighted in this rare subset of patients.

## Introduction

Lynch syndrome is a genetic condition that accounts for 2–3% of colorectal cancers and is often associated with early-onset colorectal cancer (EO-CRC). In some cases, it is associated with endometrial cancer or other malignancies, including ovarian, gastric, hepatobiliary, urinary tract, brain and skin cancers. It is genetically determined by next-generation sequencing (NGS). The disorder results from a germline variation in DNA mismatch repair (MMR) genes, specifically MLH1, MSH2, MSH6, PMS or a deletion in the EPCAM gene, and is inherited in an autosomal dominant pattern [1, 2]. This indicates that heterozygous Lynch patients generally exhibit behaviour similar to that of homozygous patients. The majority of individuals with Lynch syndrome exhibit malignancies defined by DNA mismatch repair deficiency (dMMR) and high microsatellite instability (MSI-high), which demonstrate significant responses to immunotherapy utilizing anti-PD1/PDL1 checkpoint inhibitors [3, 4]. However, little is known about the impact of immunotherapy on heterozygous Lynch syndrome colorectal cancer patients with tumours that exhibit a dMMR/MSS phenotype. Historically, advanced colorectal cancer patients with dMMR/MSS tumours are treated with 5-fluorouracil (5-FU)-based chemotherapy and fail to respond to immunotherapy [4, 5].

Here, we present a case of a MSS colorectal cancer patient and Lynch syndrome treated in our hospital and then a review of the existing literature on the application of immune checkpoint inhibitors in individuals with Lynch syndrome possessing dMMR/MSS colorectal cancer.

## Material and Methods

Following approval from the Institutional Review Committee, we examined the medical records of the Medical and Clinical Oncology Department at the University Hospital of Ioannina for patients with confirmed Lynch syndrome diagnosed with dMMR/MSS colorectal cancer up to May 2024.

We then conducted a systematic review in databases PubMed/MEDLINE, Scopus, Embase using the algorithm (lynch AND mss) AND (cancer OR adenocarcinoma) AND (colon OR colorectal OR rectal). We additionally searched in abstracts of the congresses ESMO, ESMO GI, ASCO, ASCO GI and in journals Annals of Oncology, The Lancet, The New England Journal of Medicine, ESMO Open and The ASCO Post the last 5 years. The search topic was immunotherapy in dMMR/MSS tumours when known Lynch Syndrome.

## Results

**Fig. 1:**
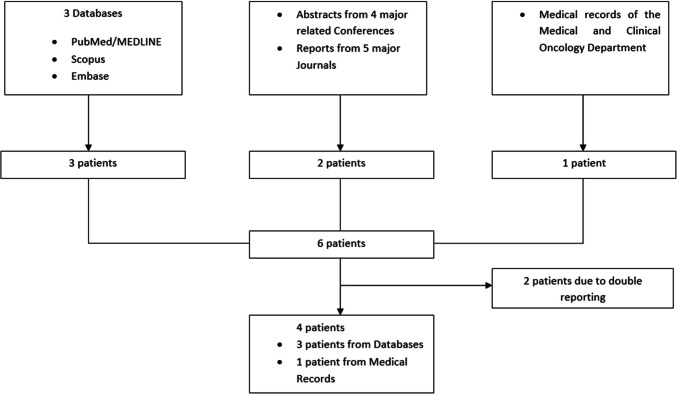
Flowchart. A systematic review of literature for patients with Lynch syndrome and dMMR/MSS colorectal cancer

Four patients with Lynch syndrome and dMMR/MSS colorectal cancer were identified (Fig. 1 — Flowchart). Patients were younger than 51 years (range 16–51 years). All four patients were found to have a germ line mutation for MSH6, but their tumours were MSS. Three individuals received immunotherapy, one as neoadjuvant treatment and two for advanced disease (Table 1).

**Table 1:** Characteristics demographics of patients with Lynch syndrome and dMMR/MSS cancers from our systemic review of literature

Patient	Source	Age	Gender	Lynch tumor gene (variant)	Setting	Cancer MSI status	Histological type	Tumor Site	ICI Drug Used	Response	Duration of response
1	Medical Records	35	Female	MSH6 (p.G1105Wfs*3)	Metastatic	MSS	Adenocarcinoma	Right-colon	Pembrolizumab	PD	3 months
2	Database PubMed	16	NA	MSH6 (c.3416delG)	Metastatic	MSS	Mucinous Adenocarcinoma	Left-colon	Bevacizumab/ Ipilimumab	PD	NA
3	Database PubMed	51	NA	MSH6 (c.741delA)	Neo-adjuvant	MSS	Adenocarcinoma	Right-colon	anti-PD-1	PR	NA
4	Database PubMed	42	Female	MSH6 (p.R495*)	Adjuvant	MSS	Adenocarcinoma	Rectal	NO(chemotherapy)	NA	NA

*ICI* check point inhibitor drug used, *MSS* microsatellite stability, *MSI* microsatellite instability, *anti-PD-1* anti-programmed cell death protein-1, *PD* progressive disease, *PR* partial response, *NA* not assessable

The patients with advanced disease showed disease progression within 3–6 months of immunotherapy treatment. The patient who received neoadjuvant treatment had a partial response without further clinical information. No complete responses were observed.

The clinical characteristics of patients treated with systemic treatment are detailed below.

The first patient was treated at the University Hospital of Ioannina. Patients 2, 3 and 4 were identified through a systematic review of the literature.

- *Patient 1*: Thirty five-year-old female patient who was diagnosed with right-sided colon adenocarcinoma.pT4 pN1a (1/19), grade II with lymphovascular and perineural invasion, R0, MSS. KRAS and BRAF were wild type. She underwent right hemicolectomy. She was treated with 8 cycles of adjuvant capecitabine/oxaliplatin. Five months later, a repeat scan revealed metastatic disease with a solitary lung lesion in the right lower lobe, and a metastasectomy was performed with clear margins (R0 resection). Histology was consistent with colon adenocarcinoma. (CDX20 + , CDX7 − , CDX2 + and TTF1 −), and MSS status was also confirmed in the specimen using immunohistochemistry. Due to her young age and a first degree relative diagnosed with cervical cancer, a next-generation sequencing of peripheral blood was conducted, and an MSH6 pathogenic gene (p. G1105Wfs*3) was identified compatible with Lynch syndrome. One month later, she was diagnosed with new liver metastases. She started treatment with pembrolizumab and after 4 cycles, the known liver metastases progressed and two new liver metastases appeared. She started FOLFIRINOX-Bevacizumab for three cycles and a partial response was observed. She was treated with FOLFIRINOX for a fourth cycle and after a multidisciplinary board discussion, it was decided that she should undergo surgery for the liver metastases.
- *Patient 2*: A sixteen-year-old patient diagnosed with left-sided metastatic mucinous colon cancer carrying a pathogenic MSH6 gene variant (c.3416delG) and exhibiting a tumour mutation burden of 151.4 mutations/Mb. The patient was treated with bevacizumab/ipilimumab and showed disease progression [4].
- *Patient 3*: Fifty-one year old patient with Lynch syndrome with a right-sided colon adenocarcinoma with a pathogenic MSH6 gene (c. 741delA), TMB data not available. The patient was treated with an anti-PD1 antibody (exact drug not reported) in the neoadjuvant setting and the interval scan showed a partial response [4].
- *Patient 4*: Forty-two-year-old female patient with two synchronous rectal tumours. The patient underwent extended radical resection of both masses. She had Lynch syndrome, a deleterious germline mutation in MSH6, abundant expression of PD-L1 and high mutation burden (TMB). One of the two masses was MSS/MSI low, had a high TMB (60.93 mutations/Mb, percentage: 99.33%) and a pathogenic MSH6 gene. She received pelvic radiotherapy and chemotherapy (three cycles of capecitabine/oxaliplatin). The patient remained relapse-free at the time of manuscript submission (April 2021). Both the patient’s mother and brother, but not her sister, were identified as carriers of the MSH6 p.R495* germline mutation, similar to the patient [5].

## Discussion

Lynch syndrome is an autosomal dominant disorder, and patients who develop malignancies exhibit tumours with an MSI-high phenotype, characterised by multiple frameshift mutations, particularly in areas of repetitive DNA sequences. These mutations are subsequently presented by HLA class I receptors as neoantigens, which engage and activate the immune system.

Malignancies in heterozygous Lynch patients typically behave similarly to those in homozygous patients, as MSI-high disease. However, people with Lynch syndrome can develop an MSS tumour. Other malignancies, including lung and ovarian cancer, are being studied in Lynch syndrome, where microsatellite stable (MSS) tumours have been noted. However, the response to immunotherapy in this subgroup has not been well studied [6, 7].

A review of the literature on this specific topic revealed a paucity of pertinent studies, with only three patients identified. This may be attributed, at least in part, to the observation that the majority of colorectal tumours associated with Lynch syndrome exhibit a high microsatellite instability (MSI) status [8–10]. However, it cannot be excluded that the population of Lynch syndrome patients with tumours that have a microsatellite stable (MSS) profile is likely to be significantly underestimated. In fact, a report from Denmark on ovarian cancer in Lynch syndrome showed that out of 26 evaluable tumours, 14 were MSS (53.8%). However, when assessed by immunohistochemistry, 93% of the cases were dMMR, indicating the lack of concordance between PCR and IHC in gynecological malignancies [7]. In the context of colorectal cancer, it has been demonstrated that up to 98% concordance exists between MSI-H and MMR-d [11].

All four reported patients exhibited a germline mutation in MSH6 and all were young, aged between 16 and 51 years. Previous studies that suggest that the median age of onset of colorectal cancer varies depending on the specific germline mutation that is present. It has been suggested that the age of onset of colorectal cancer in MSH6 mutation carriers is later than in those with MLH1 or MSH2 mutations [12, 13]. Furthermore, gender disparities may exist in the median age of onset [13, 14]. Two patients exhibited rectal tumours, while two others displayed right-sided tumours. However, the limited sample size precludes any definitive conclusion regarding the age presentation or the side preference of these tumours and their potential alignment with the right-sided predominance observed in Lynch syndrome, as documented in the literature [15]. To our knowledge, this is the first report analysing the available evidence for the use of anti-PD1/PDL1 checkpoint inhibitor immunotherapy in patients with Lynch syndrome for the treatment of colorectal cancer with MSS profile.

There is increasing evidence to support the benefits of immunotherapy in MSI-high colorectal cancer including patient with Lynch syndrome. Pembrolizumab, an anti-programmed cell death protein 1 (PD-1), showed significantly longer progression-free survival than chemotherapy as a first-line treatment with fewer treatment-related side effects in patients with metastatic MSI-H/d-MMR colorectal cancer [16]. The efficacy of immunotherapy in MSI-H colorectal cancer has been demonstrated in the preoperative setting, with a notable proportion of patients treated with nivolumab and ipilimumab exhibiting a remarkable pathological response [17]. Nevertheless, the utilization of IO in MSS tumours remains an unmet clinical requirement [18].

In the reported series of patients with Lynch syndrome, two out of three showed disease progression on immunotherapy. This is in direct contrast to Lynch syndrome patients with MSI-high cancers, in whom the response to treatment is beneficial and particularly impressive [3, 4]. The response of Lynch syndrome patients with cancer to ICI treatment appears to be determined mainly by MSI status of the primary tumour rather than by other genomic mutations associated with Lynch syndromes, and it is not surprising that these patients did not respond to ICIs.

Our study exhibited certain limitations. The medical records of the Medical and Clinical Oncology department of our hospital were examined retrospectively. The sample size was notably limited; individuals exhibited varying disease stages and did not get uniform ICI treatments. Moreover, some details about the site of metastases, the duration of response and molecular profile of the tumour such as KRAS, BRAF were not available in the included studies.

In conclusion, patients with Lynch syndrome possessing dMMR/MSS colorectal cancers are likely underreported in the literature [19], diagnosed at a young age, and the probability of these cancers responding to contemporary treatment with immune checkpoint inhibitors is exceedingly unlikely, thus should not be employed outside of clinical trials.

## Data Availability

No datasets were generated or analysed during the current study.
